# Developing templates for uniform data documentation and reporting in critical care using a modified nominal group technique

**DOI:** 10.1186/1757-7241-21-80

**Published:** 2013-11-26

**Authors:** Hans Morten Lossius, Andreas J Krüger, Kjetil Gorseth Ringdal, Stephen JM Sollid, David J Lockey

**Affiliations:** 1 Department of Research and Development, Norwegian Air Ambulance Foundation, Holterveien 24, Drøbak 1441, Norway; 2 Field of Pre-hospital Critical Care, Network for Medical Sciences, University of Stavanger, Kjell Arholmsgate 41, Stavanger 4036, Norway; 3 Department of Anaesthesiology and Intensive Care, St. Olav University Hospital, Prinsesse Kristinas gate 3, Trondheim 7030, Norway; 4 Department of Anaesthesiology, Vestfold Hospital Trust, P.O. Box 2168, Tønsberg 3103, Norway; 5 Emergency Division, Oslo University Hospital Ullevål, Nydalen Kirkeveien 166, PO Box 4956, Oslo 0424, Norway; 6 School of Clinical Sciences, University of Bristol, Bristol BS16 1LE, UK; 7 Department of Anaesthesia, North Bristol NHS Trust, Bristol BS16 1LE, UK

## Abstract

**Background:**

Clinical practice in trauma and critical care is predominantly derived from quantitative observational cohort studies based on data retrospectively collected from medical records. Such data create uncontrolled bias and influence external and internal validity, thereby hindering systematic reviews. Templates or standards for uniform documenting and scientific reporting may result in high quality and internationally standardised data being collected on a regular basis, enhance large international multi-centre studies, and increase the quality of evidence. Templates or standards may be developed using multidisciplinary expert panel consensus methods.

We present three consensus processes aimed at developing templates for documenting and scientific reporting. We discuss the advantages, limitations, and possible future improvements of our method.

**Methods:**

The template preparation was based on expert panel consensus derived through a modified nominal group technique (NGT) method that combined the traditional Delphi method with the traditional NGT method in a four-step process.

**Results:**

Standard templates for documenting and scientific reporting were developed for major trauma, pre-hospital advanced airway handling, and physician-staffed pre-hospital EMS. All templates were published in scientific journals.

**Conclusion:**

Our modified NGT consensus method can successfully be used to establish templates for reporting trauma and critical care data. When used in a structured manner, the method uses recognised experts to achieve consensus, but based on our experiences, we recommend the consensus process to be followed by feasibility, reliability, and validity testing.

## Background

Current clinical critical care practice is, to varying degrees, evidence based. Evidence-based practice is commonly derived from reports published in international registries and libraries (e.g., the Cochrane Controlled Trials Register) [1], which is primarily based on recommendations from randomised controlled trials (RCT). Although RCTs are considered the gold standard for medical research, ethical, legal, and practical aspects limit the establishment of sound RCT protocols in critical care. Critical care patients are, by definition, rarely amenable to informed consent, and there is a consequent lack of RCTs in this field of medicine [2]. The scientific reports in critical care are predominantly based on quantitative observational cohort studies and animal studies [3]. Observational studies are conducted more easily in a critical care environment in terms of ethical approval and interference with routine clinical activity. They typically require much lower funding levels. Prospective observational studies are valuable alternatives to RCTs and will continue to supply crucial scientific evidence. But commonly, observational studies are based on data derived from dedicated registries, data retrospectively collected from medical records, or (more rarely) data that are prospectively collected for the study purpose itself. The quality of data collected routinely for other purposes may be of variable quality [4,5]. Even in cases in which the data quality is satisfactory, data that are defined and collected for other purposes can create uncontrolled bias and influence external and internal validity [6,7]. Uncontrolled bias and lack of external validity can make the interpretation of results challenging [8] and hinder systematic reviews [9-11].

This point is well illustrated by the recently published ERC Guidelines 2010 for cardiopulmonary resuscitation and emergency cardiovascular care, which are substantially based on low-level evidence. Observational studies constitute a significant proportion of the reference list [12]. This process ranks RCTs highly, but they are uncommon, often inconclusive (non-significant), and rarely provide sufficient evidence to construct a robust guideline. Most of the recommendations are based on low levels of evidence.

A primary challenge to researchers and clinicians has been to improve the quality of observational data collected in day-to-day practice. One method is to develop templates or standards to uniformly document and report data. A template or standard ensures that the reported variables in specific patient groups, specific emergency medical conditions, and from specific interventions are consistent and reproducible. Such standardised variables with precise definitions may strengthen the quality of routinely collected data and the validity of published reports, thereby facilitating the analysis of reports in producing systematic reviews. High quality, well defined, and internationally standardised data that are collected regularly might enhance large international multi-centre studies and increase the quality of evidence [13].

Templates or standards for documenting and reporting data may be developed using qualitative methods, such as multidisciplinary expert panel consensus methods [14,15]. There is an acceptance and tradition for using formal consensus development methods to examine the appropriateness of clinical interventions, to develop guidelines for diagnosing and treating specific diseases, to identify education and research priorities, and to facilitate studies on preventable deaths because of problems in patient care [1,16]. Consensus development methods allow a combination of evidence-based knowledge, personal experience, and general insight into the characteristics of the patient cohort assessed or problem addressed. Critical steps following the development of templates for documenting and reporting event data is the implementation of the agreed data variables in existing registries, and securing the reliability and validity of the defined data variables.

One of the first consensus-based templates published for use in critical care was uniform documenting and reporting of data for out-of-hospital cardiac arrest, which was published in 1990 by a task force preceding a conference at Utstein Abbey, Stavanger, Norway [17]. Since then, the Utstein Abbey has hosted many consensus development meetings that have resulted in similar “Utstein Style” guidelines, such as paediatric advanced life support [18], laboratory cardiopulmonary research [19], in-hospital resuscitation [20], major trauma [21,22], disaster management [3,23], emergency medical dispatch [23], pre-hospital airway management [24], physician manned emergency medical services [25], and drowning [26]. These meetings have achieved consensus based on variants, modifications, or mixtures of the Delphi method [14], the nominal group technique (NGT) method [14], and/or the consensus development conference method [15].

In this paper, we will present and discuss the modified NGT method, which was used in three of these Utstein processes [22,24,25], and discuss the advantages, limitations and possible future improvements of the method. The three processes aimed to develop templates for documenting and reporting data for scientific purposes.

## Methods

The Delphi method has been widely used in health care research for defining priorities in education, clinical practice, organisation, and planning. It is commonly based on three e-mail rounds in which a large number of experts provide opinions on specific matters. The opinions are grouped and re-circulated for ranking, and again summarised and circulated for a re-ranking based on the individual experts' insights in the group response.

The NGT method originates from efforts in large industrial companies aiming for a more structured decision-making tool [27] The NGT methods gather a number of specifically invited experts, commonly 10–15, for a structured meeting on a specific subject [14]. The meeting is divided into separate rounds, in which the experts propose, rate, discuss, and re-rate a list of items, variables, or questions. The discussions are facilitated by an expert or non-expert who is highly familiar with the method. Consensus is reached by the end of the meeting.

### The modified nominal group technique method

The preparation of the three templates referred to in this paper was based on a modified NGT method that combines the traditional Delphi method with the traditional NGT method. The entire process consisted of four steps. The first part of the consensus process (step one and two) uses the Delphi method approach to allow the experts to identify data variables relevant for the template under development. To fully utilise the clinical and scientific competence of the experts, they are allowed to interact by applying the NGT method (step three and four).

#### Step one

In the first step, each expert was supplied with the necessary background documents (i.e., the existing templates, key papers on the subject, and clinical guidelines), which were gathered by a co-ordinating project group. The expert was asked to return (by e-mail) the proposals for inclusion and exclusion criteria, a set maximum number of core data variables in a prioritised order, and optional data variables that were regarded as important for the template preparation. The proposed variables were divided into set variable subgroups. A maximum number of core variables were defined by the co-ordinating project group prior to each process, with the intention of keeping the expert panel focused on the core data.

#### Step two

These initial proposals were aggregated and systemised by the co-ordinating project group according to the frequency with which the variable had been proposed by the experts. The collated results were redistributed to the experts for comments and the experts were asked to rank the variables within each subgroup from one to 10. The results from step two formed the basis for the expert panel meeting (step three).

#### Step three

The third step consisted of one or two consensus meetings in which the members of the expert panels, in groups and plenary sessions, discussed their views in a structured way and reached their conclusions. The consensus meeting differed significantly in structure from the e-mail rounds. During the meeting, the discussion was open, allowing interactions between the panel members to influence the ranking and conclusions, including novel variables if agreed upon. Exceeding the set maximum number of variables was allowed pending group approval.

#### Step four

In the fourth step, based on the conclusions from the consensus meeting(s), the co-ordinating project group edited a final proposal for a template, upon which the experts were allowed to comment by e-mail. To complete the process, a letter of agreement was signed by all the expert panel members to enhance the implementation of the achieved template in the daily documentation of practice.

### The experts

Three international expert panels were selected to participate in these three Utstein processes. Because of the structure of the consensus meeting, a maximum number of twenty experts per process were set. As the experts, we invited senior researchers and clinicians who had contributed substantially to research; the development of guidelines, existing registries, and/or clinical practice; and were considered experts in the specific field of critical care for which the template was developed. The experts were identified by Google and PubMed searches on the subject, through personal networks of the co-ordinating project group, and by recommendations from previously selected members. The expert panel was invited by e-mail and personal contact, and all were asked to include information from their own experience or knowledge in the process. The invited experts who could not attend were asked to suggest a substitute colleague. Three reminders were sent to the non-responders.

### The e-mail rounds

In the e-mail rounds, the experts were supplied with a spreadsheet that was designed as a template for the proposals. The template was divided into category subgroups of data determined by the purpose of the template, e.g., system variables, patient variables and process variables. Each variable required additional information on the exact data variable definition, the possible data variable categories, and the data variable source (e.g., hospital record, EMS record).

After each round, the experts returned their completed spreadsheets of proposals to the co-ordinating project group.

### The consensus meeting

In step three of the modified NGT method, the expert panel gathered at a two-day meeting and agreed on the inclusion and exclusion criteria and a core data set for the template. Two experienced scientists and clinicians who were familiar with the method facilitated the meeting of each of the three Utstein processes. In a first plenary section, the co-ordinating project group presented the proposed variables from step two, and the facilitators presented the set structure for the meeting. The experts were divided into two groups and separately discussed the specific inclusion and exclusion criteria and variables for the proposed dataset. The groups subsequently presented their discussions in plenary sessions, at which all variables were discussed, debated, and agreed upon. On day two, the variables were given precise definitions and categorised in a plenary session. In two of the consensus meetings [7,24], the project group allowed a few variables to not be accompanied by specific definitions during the meeting, authorising the co-ordinating project group to propose final definitions to be decided during step four of the consensus process.

## Results

The expert panels achieved consensus during the planned four steps in all three Utstein processes. The structure of each process differed slightly.

### Documenting and reporting data for severely injured patients

Twenty-three experts were invited, and 19 accepted the invitation and joined the Utstein process. In 2007, the expert panel was asked to propose inclusion and exclusion criteria, as well as a maximum of 30 core data variables in a prioritised order. The first proposals were summarised and structured by the co-ordinating project group, and the collated results were redistributed in step two for comments and re-prioritisation. In step three, the expert group did not reach consensus on all variables because of extensive discussions and a prolonged decision process. To finalise the process, the expert group decided to conduct a second consensus meeting. During these two consensus meetings, which were held at the Utstein Abbey in May and December 2007, the panellists discussed their views by a structured method and reached consensus.

Of the 19 individuals participating in the e-mail process, 18 participated in the first consensus meeting and 16 in the second meeting. No formal communication regarding this process occurred among the experts between the two meetings. After completing the process, the expert panel had agreed upon the inclusion and exclusion criteria, 36 core data variables and four subsidiary variables for the template. The data variables were divided into three groups: ‘Predictive Model’ variables, ‘System Characteristics Descriptors’, and ‘Process Mapping’ variables. Each expert signed a letter of consent (agreement), and the results were published in an international, scientific, open access journal in July 2008. The co-ordinating project group published a data definition catalogue on a dedicated web site with open access.

### Documenting and reporting in pre-hospital advanced airway management

In January 2009, 15 experts accepted invitations to join the Utstein process. The panel was asked to propose 10 core data variables and five optional variables in a prioritised order. The first proposals were summarised and structured by the coordinating project group, and the collated results were redistributed in step two for comments and re-prioritisation.

During the consensus meeting at Utstein Abbey in April 2009, the expert panel agreed that any patient receiving advanced airway management, defined as the attempted insertion of an advanced airway adjunct or administration of ventilatory assistance, should meet the inclusion criteria. The expert panel agreed that advanced airway management during inter-hospital transfer should be excluded. The expert panel agreed on 23 core data variables that were divided into three groups: system, patient, and post-intervention. Each expert signed a letter of consent before the results were published, and the results were published in an international, scientific, open access journal in November 2009.

### Documenting and reporting in physician staffed pre-hospital EMS

In winter 2010, an expert panel was invited and asked to propose inclusion and exclusion criteria, as well as a maximum of 50 core data variables in a prioritised order. Seventeen experts were invited, and 16 accepted the invitation and joined the Utstein process. Their first proposal was summarised and structured by the coordinating project group, and the collated results were redistributed in step two for comments and re-prioritisation. In step three, the expert panel met and decided on the definition of physician staffed pre-hospital EMS, inclusion criteria, and 50 variables that were divided into five subgroups: “fixed system variables”, “event operational descriptors”, “patient descriptors”, “process mapping”, and “outcome measures/quality indicators”.

The final core data set was sent to the experts by email after the meeting, and the experts were allowed to make comments to the final data set. A few minor changes, which were mainly related to data definitions, were made at this point. When no more comments were received, the consensus process was formally closed.

The results were published in an international, scientific, open access journal in November 2011.

## Discussion

Most critical care services document their activities daily, but the documentation is typically based on local data definitions and patient categorisations. The difficulty of comparing trauma and critical care between centres and over time based on locally defined data variables has been illustrated in several recent reports [6-8,28]. Over the last two decades, several templates for standardised documentation and reporting in critical care have been published. These templates have proven valuable, particularly in the field of cardiac arrest research, in comparing the activity, effect, and efficiency of health care systems.

Traditionally, such templates have been designed using consensus methods. In this paper, we present a modified NGT method used in three consensus processes with the aim of developing templates for reporting from three specific areas of trauma and critical care.

We perceived a number of benefits from the use of this modified NGT method to reach consensus. A consensus process derives its credibility, in part, from the composition of the expert panel. In these three consensus processes, the experts were professional authorities who were key stakeholders in their services and respected representatives of their profession. They had significant scientific credibility within their fields of medicine. The initial proposals from the experts were unconfined by the group dynamics. In step two, the individual experts had access to all proposals and could review their suggestions in comparison to those of the other respondents. The processes were controlled, giving authority and rationality to the conclusions made by the expert panels. The conclusions were based on pre-existing knowledge introduced to the process partly by scientific papers and reports identified by the co-ordinating project group prior to the process and partially by the competence and experience of the individual experts. Each proposed variable was thoroughly discussed face to face with the opportunity to include new proposals that were not included in the e-mail round (steps one and two). There is a high likelihood that the structure of the process secured the capture of all the collective knowledge on the subject at hand. Upon reaching consensus, the majority of the experts could, empowered by their position, implement the template in daily documentation of practice.

A particular challenge during the consensus process was defining the word *consensus.* Methodologically, there is no convention for the exact definition of consensus, but some measure of agreement in the panel was critical. In consensus processes, defining a few elements, such as quality indicators or focused research areas, as a statistical measurement of agreement is feasible. In our process, we aimed to agree upon many different variables (i.e., a data set) and the data set definitions. Therefore, by applying a voting strategy in our process, the voting would have taken too much time and hindered fruitful discussions amongst the panellists. Thus, we defined consensus as no objection to the final dataset, including definitions, by the expert panel members.

### Limitations

There are obvious limitations to our method. The choice of experts was partly unstructured and did not fully guarantee a representative selection of experts within all the subfields of the subject at hand. This design potentially leads to the omission of vital competences and the omission of important variables. NGT processes may be vulnerable to collective group ignorance caused by the risk of dominance by expert panel members. We hypothesised that the multi-national selection of experts with fairly detailed and predefined selection criteria reduced the risk of establishing an expert panel with biased opinions.

The project group decided to provide the expert panel members with a selection of background literature. The experts were advised to focus on the existing templates. The developed templates are defined and established by experts within the specific field of care and should be acknowledged as relevant. The possibility of this “literature-based” bias appears minimal given the extensive amount of different variables proposed in the first rounds.

In contrast to the Delphi method, the NGT method may be more prone to the possibility that strong personalities can dominate the group. The NGT method does not secure anonymity for the participants, as does the Delphi method. Maintaining proposals as anonymous during the expert panel processes may be important to reduce influence of “loud-speaking” experts and to facilitate the influence of their “silent-speaking” peers. The expert panel members were aware of its composition, but anonymity related to the proposals was maintained until stage three.There is no evidence that the implementation of standardised templates for documenting and reporting will lead to appropriate standardized practices, and hence this was not a purpose of these consensus processes. The process does not test or check the true feasibility of the template or the validity and reliability of each variable; instead, it reflects the collective conviction of the expert panel. Expert opinion will fluctuate due to new evidence and knowledge. A template developed by the described technique is only as good as the available evidence, and future revisions may significantly change the recommendations.

### Future improvements

The experiences from these three consensus processes suggest possible improvements. The recruitment of experts should be structured and formalised. Our recruitment strategy was based on unstructured PubMed and Google searches and personal professional networks. To reduce a potential bias, a recruitment strategy should be based on a defined search strategy and/or pre-defined specific qualification criteria. To ensure that all available evidence is included in the process, it should be preceded by a systematic search for evidence. The quality of evidence of the findings from the search should be graded before use in the consensus process.

The development of templates for standardised documenting and reporting is a dynamic process and following the consensus process, feasibility, validity and reliability studies should be performed. This process includes a pre-test or feasibility study of the template to identify problems in applying the template to subjects in the clinical setting. The template should be pre-tested to assess its ease of use and acceptability to its administrators and targeted population [11]. Typically, a feasibility study that involves trialling a dataset/data collection tool will not give a true indication of how easily the data variables are collected if it is only trialled in services or hospitals that are committed to high quality data collection. A broad range of relevant institutions should be recruited for feasibility studies. In cases of data collection difficulties, the data variables, response categories and definitions may be improved or excluded. Following the publication of the Utstein Template for Uniform Reporting of Data Following Major Trauma (Utstein Trauma Template), we performed a study on the feasibility of collecting core data from registries that had implemented the template [29], which provided important feedback to a revision process.

There are a number of methods of assessing a template’s validity. Content validity, construct validity, and criterion validity should be tested [11,17,18,30]. Content validity explores the relevance and completeness of the content of a tool and can be considered a part of our described modified NGT method. Construct validity refers to the extent to which application of the template or the single data variable actually measures that which they intended (e.g., to what extent does an IQ questionnaire actually measure "intelligence"?). Criterion validity involves a comparison of the single data variable assessment with that obtained using a gold standard procedure. If the data variable performs well, good agreement is expected between the assessments, as reflected by a high level of criterion validity [18,30]. To measure the criterion validity of a tool, researchers must calibrate it against a known standard or against itself. After publishing the Utstein Trauma Template, we performed a study of the criterion validity of one core data object, the Abbreviated Injury Score (AIS) [31], which was scored by trauma registrars [32]. The assessment of a tool’s validity is an on-going process, and several studies from different institutions may be necessary to investigate the construct validity of the data collection and rating tools.

The reliability of the proposed data variables should be evaluated by measuring the agreement when applied by different users. This concept is known as inter-rater reliability or inter-observer agreement [11,17,30]. Poor reliability or performance of a tool can be caused by several factors, including rater-experience, template limitations, and database limitations. After implementing the Utstein Trauma Template, we tested the reliability of the AIS, as scored by trauma registrars [32], and the pre-injury physical status classification, according to the American Society of Anaesthesiologists Physical Status, was tested [33]. In the first paper, the reliability was poor to mediocre [32]. Improvement in agreement may be achieved by more precise definitions of the data variables, a reduction in the number of AIS codes, and more experienced raters.

Reliability places an upper limit on validity (i.e., higher reliability follows higher maximum possible validity) [30]. Because the level of a variable's reliability influences its validity, reliability studies should be performed prior to validity studies [18].

The implementation of consensus developed reporting templates in day-to-day documentation is the major weakness in achieving a common platform for intra- and inter system comparison.

With key stakeholders involved, the Utstein Trauma Template was implemented as the standard reporting method by hospitals to the UK Trauma Audit and Research Network, the Trauma Registry of the German Society of Trauma Surgery, and the Italian National Registry of Major Injuries. A number of hospitals and registries in Scandinavia and central and southern Europe implemented the core dataset. This wide usage resulted in more than 500 hospitals implementing the template. In the two remaining consensus processes, we have not achieved the identical degree of implementation despite a number of strong recommendations [13] and implementation in certain pre-hospital EMS systems.

### Future implications

Several studies have focused on the intricacy of implementing advanced medical interventions in the pre-hospital setting [34-36]. Advanced medical interventions commonly represent a *complex intervention* containing several separate, but highly interacting components. Scientific studies on this subject are difficult to design and interpret because of tremendous variability in (and insufficient description of) operator experience, technique, and patient case-mix, making it difficult to understand or eliminate confounding factors [37]. Continous documenting and reporting from such interventions based on a template developed by expert panel consensus may cover contemporary interventions and pre-, per- or post-intervention factors highly likely to influence outcome. The template will then not only enhence outcome analysis, but also allow detailed system comparisons and individual system factors relation to outcome. In our opinion, expert panel consensus based templates will be a good instrument for gathering documentation to analyse the effect and feasibility of specific advanced pre-hospital emergency medical interventions.

## Conclusion

It is hypothesised that standardised data reporting from health care systems could enhance inter- and intra-system comparisons. We conclude that a modified NGT method can successfully be used in consensus processes to establish templates for reporting trauma and critical care data. If used in a structured manner, the modified NGT can achieve consensus among recognised experts, but based on our experiences, we recommend that a consensus process is followed by feasibility, reliability, and validity testing.

## Competing interests

The authors declare that they have no competing interests.

## Authors’ contributions

HML, AJK, KGR, SJMS and DJL have made substantial contribution to the conception and design of the presented method, and the analysis and interpretation of data. HML drafted the manuscript, which was finally approved by all authors.
